# Assessment of Ambivalent Sexism in University Students in Colombia and Spain: A Comparative Analysis

**DOI:** 10.3390/ijerph18031009

**Published:** 2021-01-24

**Authors:** Aura Yolima Rodríguez-Burbano, Isabel Cepeda, Ana Magdalena Vargas-Martínez, Rocío De-Diego-Cordero

**Affiliations:** 1 Program of Law, Faculty of Social, Political and Humanities, Universidad de Santander, 680003 Bucaramanga, Colombia; 2 Applied Economy Department, Rey Juan Carlos University, 28032 Madrid, Spain; mariaisabel.cepeda@urjc.es; 3 Nursing Department, School of Nursing, Physiotherapy and Podiatry, University off Seville, 41009 Seville, Spain; rdediego2@us.es

**Keywords:** sexual education, knowledge of sexuality, ambivalent sexism, university, gender-based violence, sexism

## Abstract

(1) Background: Gender-based violence has no geographical, personal, or social boundaries. It constitutes a serious public health problem that affects the entire society. This research aims to identify and compare the level of ambivalent sexism in Spanish and Colombian university students and its relationship with sociodemographic factors. Ambivalent sexism, developed by Glick and Fiske (1996), is considered a new type of sexism since, for the first time, it combines negative and positive feelings that give rise to hostile and benevolent sexism, maintaining the subordination of women through punishment and rewards. (2) Methods: The methodology consisted of the application of the validated Spanish version of the Ambivalent Sexism Inventory (ASI) to a sample of 374 students in their final academic year of the Law program, of which 21.7% were students at the University of Santander (Bucaramanga, Colombia), 45.5% at the University Rey Juan Carlos (Madrid, Spain), and the remaining 32.9% at the University of Seville (Seville, Spain). (3) Results: A high level of ambivalent sexism is reported in Colombian students nowadays. In the two countries. there are similarities (e.g., the great weight of religion and the variation in attitudes towards sexism in people who identify themselves as women, compared to male or students consulted that prefer not to answer) and differences (e.g., absence in Colombia of gender-specific legislation, low number of students who have received gender education in Spain). (4) Conclusions: These findings may contribute to the construction of laws that take into account the particular problems of women and the development of educational programs on gender that are offered in a transversal and permanent way and that take into account cultural factors and equity between men and women as an essential element in the training of future judges who have the legal responsibility to protect those who report gender violence.

## 1. Introduction

Gender-Based Violence (GBV) is violence directed against a person because of that person’s gender or violence that affects persons of a particular gender disproportionately and is a serious public health problem, defined as abuse of women by virtue of their gender identity, most often perpetuated by men’s control over women through physical, emotional, financial, and/or sexual means [1,2]. In January 2016, about one in three women worldwide (35%) had experienced either physical and/or sexual violence by their partner. In Europe, more than 25 million women experienced psychological, physical, and/or sexual abuse in 2015 [3]. As for Colombia, in 2019, it was estimated that “one woman is killed by her current or former partner every three days, at least one woman is assaulted by her current or former partner every 13 min, and one woman is sexually assaulted every 24 min” [4]. In Spain, in the same year, 31,911 women were victims of GBV, of which 55 lost their lives due to this type of violence [5]. Considering that these statistics are often underestimated [6], it becomes clear that we are facing a major social situation.

According to Ina Kenner, sexism was initially used in the 1960s in connection with racism [7,8]. In the same vein, Marta Lamas [9] refers to sexism as “sex-based discrimination.” As a “venerable institution”, sexism refers to the subordination of women. Sexism is mostly a problem of women in relation to men. For Lameiras [10], sexism is “an attitude aimed at people on the basis of belonging to a certain biological sex for which different characteristics and behaviors are assumed”. This concept has undergone several transformations from the so-called old sexism—traditional sexism—[8] to the new sexism—neosexism—[10,11], in which Ambivalent Sexism is emphasized [12]. Ambivalent Sexism is made up of two components: Hostile sexism and benevolent sexism, which although different, may converge to a certain extent. The first concept is based on the presumed inferiority of women inherent to traditional sexism, which is supported by three postulates: The inferiority of women, the lack of capacity in women to develop a role in a different environment than the private ones, and “sexual hostility”. The second concept is based on the kind of prejudice that considers women as worthy of protection, which rest on three elements: The necessary protection that should be provided to women, the complementary nature of women in relation to men’s roles, and “the dyadic dependence of men on women” [13]. These types of sexism are generally based on the domination of men over women, which is reinforced through the implementation of a system that uses elements of hostile sexism (punishment) and benevolent sexism (rewards) [12,14]. As García et al. [15], point out, this whole set generates a direct relationship between ambivalent sexism and gender-based violence. Specifically, the literature has found that people with high levels of hostile sexism have shown more positive attitudes towards violence against women than those with lower scores for this type of sexism [16].

Gender-based violence has penetrated various social environments, including universities. In Colombia, as noted by Quintero [17], very few studies are available on gender-based violence in universities, which seems to suggest that although this problem has long been present, it has been overlooked by various social mechanisms [18]. On the other hand, in Spain, the relationship between gender-based violence and universities has been analyzed for some years now. One of the first studies was carried out by Larena and Molina [19]. It highlights the importance of this area of study and recognizes the university as an environment that is not immune to gender-based violence as well as an institution that has its own hierarchical structure promoting unequal relationships. On an international scale, it is worth mentioning the studies carried out in the United States, especially the research conducted by Makepeace [20] that analyzes, for the first time, the violence experienced by college women in their relationships. It is also important to mention, especially for the research topic analyzed here, the research carried out by Kalof [21] in which it is suggested that many victims of gender-based violence in the academic environment do not perceive GBV as such, in addition to the existing relationship among the structures that reinforce a model of masculinity in which some men may have assumed. Several current studies in this field have made it possible to examine in greater depth the university as a social space that also has to deal with gender-based violence and that has special characteristics that need to be analyzed to understand the complexity of this social problem [22,23,24,25,26,27].

Gender violence’s pervasiveness as a universal public health issue is encouraged not only by sexism. Certain socio-political and cultural factors—archaic traditions, religion, lenient legislation—might also strengthen patriarchal notions of gender hierarchy. Specific gender legislation, the application of the gender perspective in university programs, or the creation of specific courts to hear cases of violence against women should contribute to countervail these sociopolitical and cultural factors and, consequently, to reduce sexism. Therefore, it is relevant to conduct studies involving the measurement of sexist attitudes in college students which, as previously mentioned, could become one of the causes of GBV. Researching among different countries can provide useful information to help develop preventive strategies to reduce GBV, such as training activities or especial education. Some studies have demonstrated that students attending gender-related courses become aware of gender issues and identify specific gender-based violence situations. They also gain a critical vision of their own reality, which undoubtedly contributes to the prevention of both gender discrimination and inequality among women [28,29,30].

To measure ambivalent sexism, Glick and Fiske [12] developed a scale in 1996 that they called the Ambivalent Sexism Inventory (ASI). The objective of the ASI is to measure hostile and benevolent attitudes towards women. The ASI consists of 22 items. Eleven of them are related to the BS and the other 11 items refer to the HS. In the questionnaire, the items related to the HS and those that refer to the BS appear mixed. Some of the items on BS are, for example, the number 3: “In the event of a disaster, women must be rescued before men”; or the number 9: “Women must be loved and protected by men.” Some items related to the HS are, for example, the number 5: “Women are very easily offended”; or the number 10: “Most women do not fully appreciate all that men do for them.” The ASI is not the only scale to try to assess sexism. Among others, the Scale for Detection of Sexism in Adolescents (DSA) [31], the Inventory of Distorted Thoughts on Women and Violence (IDTWV) [32], or the Social Desirability Scale (SDS) [33] also try to measure sexism. To carry out our study, the ASI was chosen because of the advantages it offers over the rest. DSA, for example, is aimed at detecting ambivalent sexism in non-adults, so it is not appropriate for this study. The DSA scale is easier to use with samples of adolescents. On the other hand, the items of the ASI scale preferably focus on the study of sexist attitudes in adults so they focus on issues such as work, competition, feminism. Regarding the IDTWV, it was designed for the one-dimensional assessment of the cognitive biases that violent men express against their partner. Finally, the SDS was not fit to our purpose due to the low discrimination rates of many of its items [34]. The ASI has, however, some disadvantages. First, compared to the DSA scale, whose items were obtained from the literature and from the direct experience of professionals in the prevention of GBV [31], ASI’s items were developed anew by Glick and Fiske; second, the DSA scale has the advantage of showing more clearly than the ASI scale whether there is a higher degree of BS in women than among men [31]. In any case, one of the clear advantages of the ASI scale is, as Glick et al. [35] shows, that it lends itself to a possible application in different cultural contexts since their study includes a sample of 19 countries with hostile and benevolent components of sexism in all cultures and environments.

This research aims to analyze and compare the level of ambivalent sexism in university students at the University of Santander (Bucaramanga, Colombia), the University of Seville (Seville, Spain), and the University Rey Juan Carlos (Madrid, Spain). Studying these two national cases will provide an assessment of the relationship between ambivalent sexism and socio-demographic factors.

## 2. Materials and Methods

A quantitative methodology was used in this research. The Ambivalent Sexism Inventory (ASI) [12] was applied here as it is the only instrument that addresses both aspects of benevolent sexism and hostile sexism [30].

### 2.1. Study Design

The research was a descriptive, cross-sectional, multicenter study including three universities from two countries to compare prevalence.

### 2.2. Research Protocol

To ensure acceptance, students were personally contacted and invited to voluntarily participate, excluding those who deliberately refused the offer. After explaining the purpose of the study, a self-administered, individual, and anonymous questionnaire was provided to students.

### 2.3. Population and Sample

The intervention was conducted in the Bucaramanga campus at the University of Santander, Colombia (UDES), the Vicálvaro, Alcorcón and Móstoles campuses at the University Rey Juan Carlos, Spain (URJC) and the University of Seville, Spain (US). The participants were students in their final year of the Law program enrolled in the A-2019 term (first semester of the 2019/2010 academic year). Non-probability convenience sampling was conducted with 374 students, of which 21.7% are students at the UDES, 45.5% at the URJC, and the remaining 32.9% at the US.

### 2.4. Procedure

The Ambivalent Sexism Inventory (ASI) [13] was administered to the participants in its Spanish version, which was adapted and validated by Expósito et al. [13]. This instrument has 22 items on ambivalent sexism (α Cronbach = 0.90), which is made up of two scales: The first scale contains 11 items referring to hostile sexism (α Cronbach = 0.89) (i.e., *“Young men should exert control over who their girlfriends interact with”*), and the second scale contains the remaining 11 items related to benevolent sexism (α Cronbach = 0.86) (i.e., *“A boy will feel incomplete if he is not dating a girl”*). The item response format is based on a Likert-type scale with response options ranging from 0 (“strongly disagree”) to 5 (“strongly agree”), where higher scores represent higher levels of sexism. See Appendix A and Appendix B.

Sociodemographic variables such as age, gender, ethnicity, country, and religious affiliation were measured. The variable “age” was centered using the mean since the value of 0 was not included in the study sample. In relation to the “religious identity”, this variable initially was composed by the following categories: “Hindu”, “Evangelical or Protestant”, “Catholic”, “None and I don’t believe in God”, “None, but I believe in God”, and “Other”. Due to the low frequency in most of its categories, firstly this variable was recodified in four categories to describe the sample: “Catholic”, “None and I don’t believe in God”, “None, but I believe in God”, and “Other (Hindu, Evangelical or Protestant, etc.)”. Finally, this variable was dichotomized to carry out the regression analyses giving the value of 1 to the category “Catholic” and the value of 0 to “others”, which resulted from merging all the remaining categories. Similarly, the variable “marital status” was recoded to dichotomous (1 = single; 0 = other) given the low frequency of the other response categories such as “widower”, “separated or divorced”, and “common law or common-law partnership”. All of them were unified in the category “other”.

### 2.5. Analysis

A descriptive analysis of the students’ socio-demographic characteristics was carried out by reporting these data through mean and standard deviation for quantitative variables, and frequencies and percentages for qualitative variables. The normal distribution of the total score of the ASI scale, which measures ambivalent sexism and its subscales, as well as hostile sexism and benevolent sexism, was tested for the total sample and based on the university at which the student is enrolled, by means of the Kolmogorov–Smirnov normality test. Homoscedasticity or equal variances of the ASI scale and subscales based on the university was tested by using Levene’s test. The Student’s *t*-test was used for comparison of means between the score in the total ASI scale and its subscales according to the student’s university (UDES, US, and URJC). To compare the different sociodemographic characteristics of the sample by subgroup according to the university at which the student was enrolled, the chi-squared test, student’s *t*-test, and ANOVA test were used. Factors associated with ambivalent sexism, hostile sexism, and benevolent sexism were analyzed using multivariate linear regression. The accepted level of statistical significance was 5% (*p* > 0.05). All analyses were performed using R 3.6.3 software (Free Software Foundation’s GNU General Public License: https://www.r-project.org/about.html).

## 3. Results

### 3.1. Sample Characteristics

The sample comprises a total of 357 students in the UDES (20.73%), URJC (45.10%) and US (34.17%), since 9 subjects were removed due to gaps in the questionnaire and 8 subjects did not consent to use of reported data for research (see Table 1). Nearly 66% of the total sample were identified themselves as male, having similar percentages in each university ranging from 58.1% in the UDES to 68.3% in the URJC. The average age was 22.27 ± 3.57 years, being higher and significantly different in the case of UDES from Spanish universities, and more than 4/5 parts reported to be single in all subgroups. As for religious identity, there is a higher tendency to have any religious belief among UDES students than between URJC and US students, with a difference in the last-named group between those who identified with the Catholic religion, with more than half of the students in the case of the US, which is very similar to the UDES figure, but less than a third of URJC students. Regarding family characteristics, household income was classified as middle level at about 80% of all universities, observing a difference between the US and URJC and UDES in the percentage of students who reported having a high household income. In line with student’s family situations, it is noteworthy that UDES students reported having children more often, as well as their parents being separated or divorced, than at URJC and US. Lastly, among the variables studied, in terms of gender training courses, the UDES also stands out with 66.7% of the students who said they had received such training, while the percentages in the URJC and the US were 34.4% and 44.3%, respectively. Among the characteristics previously mentioned, there were significant differences in age, religious identity, family situation, and gender training courses, depending on the university.

As for mean scores on the total ASI scale, the total sample of students obtained a mean score of 50.23 ± 22.90, finding statistically significant differences (*p* = 0.000) according to the university at which they studied. Mean scores on the subscales (hostile sexism and benevolent sexism) in the total sample were 26.53 ± 12.73 and 23.69 ± 11.32, respectively. When comparing the mean scores of the subscales for students at the different universities, these were higher in each case for students at the UDES. These differences were statistically significant with respect to the other universities (*p* = 0.000). It should also be noted that the differences in both the total ASI and subscales were statistically significant between the UDES and the Spanish universities (URJC and US), but not among the Spanish universities, except for the scale measuring hostile sexism, in which statistically significant differences were also found between the URJC and the US (see Table 1).

When analyzing the comparison of means between the total score of the ASI scale and subscales, as well as between ASI items by participant’s university, statistically significant differences were found in all items of each subscale, except for item 2 “Many women are actually seeking special favors, such as hiring policies that favor them over men, under the guise of asking for “equality” “and item 19 “Women, compared to men, tend to have a superior moral sensibility” (*p* = 0.057 and *p* = 0.053, respectively) (see Table 2). In the latter, although no statistically significant differences were found among the three universities in general, the difference between UDES and URJC did reach statistical significance. These differences found in most items are generally due to differences between the UDES and Spanish universities, but not between the latter, i.e., URJC and US. Differences were only found in item 7 “Feminists are not seeking for women to have more power than men”, item 16 “When women lose to men in a fair competition, they typically complain about being discriminated against”, item 18 “There are actually very few women who get a kick out of teasing men by seeming sexually available and then refusing male advances”, and item 21 “Feminists are making entirely reasonable demands of men.” In each of these items, the average score was higher among UDES students.

### 3.2. Factors Associated with Ambivalent Sexism, Hostile Sexism, and Benevolent Sexism

In relation to the factors associated with ambivalent sexism in the total study sample, the first univariate regression analysis found that age, gender training, identifying themselves as males or not answering in relation to gender identity, and having some belief as opposed to not identifying themselves with any religion or believing in God, turned out to be risk factors for sexism since students who met these criteria scored higher than those opposed to equality of the other variables. This analysis also found protective factors for ambivalent sexism to be being single compared to any other marital status, having any family situation other than “having children”, and being a student at a Spanish university compared to UDES. After the multivariate regression analysis, the factors that were statistically significantly associated with ambivalent sexism were age, identifying themselves as males or not reporting their gender identity, identifying themselves with any religious belief, and being a student at a Spanish university, which kept the same direction of the association studied in the univariate analysis. After completing the well-known “backward stepwise regression”, the model providing the best explanation of ambivalent sexism for the total sample, accounting for 41.35% of this variable, was composed of age, gender identity, religious identity, and home university. The average score according to the ASI questionnaire in ambivalent sexism is 55.35 for a 21-year-old URJC student who identifies himself as male and does not identify himself with any religion, even though he believes in God. This student would score an average of 58.48 if compared with another student with the same profile, except that he is a US student, which would score 3.13 points more than the URJC student with equal variables. Similarly, if the average score were calculated for a UDES student with the same characteristics as mentioned above, he would score an average of 71.52. This means that the UDES student scores 16.17 points more than a URJC student and 13.04 points more than a US student (see Table 3).

As for hostile sexism, after a multivariate regression analysis for the total sample, the associated factors were gender identity, religious identity, and home university. Among these, being a student at a Spanish university could be considered a protective factor for hostile sexism, while identifying themselves as males or not disclosing their gender identity with any religion or believing in God as opposed to not identifying themselves with any religion were found to be risk factors. On the other hand, benevolent sexism, in addition to the above factors associated with hostile sexism, was also associated with age and household income level. Both were found to be risk factors for benevolent sexism, since the older the student, the higher the score on this subscale, while the student with a high or very high household income level scored higher compared to a low or very low-income level (see Table 4).

### 3.3. Factors Associated with Ambivalent Sexism, Hostile Sexism and Benevolent Sexism Based on the University

The factors associated with ambivalent sexism in the sample of UDES students, using a multivariate regression analysis adjusted for all the variables studied, were household income level and religious identity, scoring higher among students with a high or very high household income level compared to those with a low or very low level, and those who identified themselves as having a religion other than Catholic compared to non-believers. For URJC and US, the factors associated with ambivalent sexism were gender identity and religious identity. In both Spanish universities, those who identified themselves as males or who preferred not to disclose their sexual identity scored higher than those who identified themselves as females. Similarly, as for religious identity, URJC students who did not identify themselves with any religion but still believed in God and those who identified themselves with Catholicism scored higher than non-believers. Similarly, students at the US who identified themselves with Catholicism scored higher than non-believers (see Table 5).

As for hostile sexism, it was only associated among UDES students with religious identity, while in URJC, it was associated with gender identity and religious identity, and in the US it was associated with gender identity, religious identity, and family situation. Their association was the same as in the case of the ambivalent sexism study (see Table 6).

Benevolent sexism was associated at the UDES only with religious identity, as in the case of hostile sexism. Among URJC students, benevolent sexism was statistically significantly associated with gender identity and religious identity. In addition, in the case of the US, it was associated with gender identity, household income, and religious identity (see Table 7).

It should be noted that the model used to explain the hostile sexism in the sample of UDES students was not statistically significant, so other explanatory variables should be studied. In the sample of URJC and US students, the model used to explain hostile sexism accounted for 33.33% and 43.22%, respectively, while the model used to explain benevolent sexism accounted for 32.74% and 36.35%, respectively.

## 4. Discussion

Numerous studies on sexism and its determinants are available in the literature. Generalized sexist beliefs could be one of these determinants. Although the inner psychological mechanisms that transform sexist attitudes into violent action still remain to be discovered, several studies that have been carried out in different countries on Ambivalent Sexism have concluded that both types of attitudes (BS and HS) are negatively associated with national indicators of gender equality [34]. That is, the higher the average levels of sexist attitudes in a nation, the lower this nation scores on measures of gender equality. The measurements obtained with the ASI suggest that both hostile and benevolent sexism present significant correlations with attitudes that seek to legitimize —or, at least, make more acceptable—abuse towards women within marital relationships in societies as different as Turkey or Brazil [36]. Similarly, a study carried out among 280 Spanish university students [37] showed that students who received specific gender education express greater rejection to all forms of sexism. The results suggest that gender education in the college environment contributes to making sexism and GBV visible among young people, positively influencing their value structure, and helping to triggering greater rejection of sexism and GBV. It could be argued that literature suggest that sexism and the sexist beliefs are related to gender-based violence. Considering the results obtained in this study, it is confirmed that in terms of attitudes of ambivalent sexism and the participants’ sex, there are variations in behavior towards sexism in people who identify themselves as females, compared to males or students consulted that prefer not to answer. For the Colombian university, this difference can be interpreted as a definition of the lower position of women with respect to sexism in general. As for the Spanish universities, this difference can be interpreted as a lower inclination of women towards attitudes typical of hostile sexism [29,38,39,40].

Religion is another of determinants of sexism. It is noteworthy that the sexism-religious belief relationship shows a positive sign for all universities and in all indicators, which means that those who harbor religious beliefs tend to be more sexist than those who do not. Regarding the relationship between religiosity, alcohol consumption, and predictive sexist behaviors of violence, previous studies have established an inverse relationship between the first two variables, that is, higher levels of religiosity are related to lower rates of alcoholism [41]. However, regarding the relationship between religiosity and intimate partner violence (IPV) higher levels of religiousness are associated with higher (IPV) levels. Furthermore, religious restraint was positively associated with hostile sexism in this study with Americans [42].

Taking into account the gender education variable, it should be noted that even though the results indicate that in Colombia more students have received training in gender, it seems that for these students it is not a protective factor against sexism. It should be noted here that although it is true as already indicated, the literature shows that there is an inverse relationship between sexism and gender education, it should be taken into account that in Colombia gender education is not mandatory and even when there are some efforts to introduce a chair of gender studies in undergraduate curricula [43], its application so far is of little intensity. This leads us to point out that one should think about educational programs on gender that are offered in a transversal and permanent way, taking into account cultural factors at the regional and national level.

On the other hand, regarding the family variable, the results reported that the presence of other problems at home does not imply a higher level of sexism. Moreover, any family situation other than “having children” is a protective factor against ambivalent sexism. In fact, one of the major determinants of sexism is the family given its enormous importance in transmitting values and beliefs. Some of the studies exploring how the family contributes to the gender socialization and sexist beliefs of adolescents can be found in Garaigordobil and Aliri [44], who analyze the family’s influence on the perpetuation of sexist values and attitudes, focusing on the incidence of socioeconomic level. Their findings suggest that the higher the socioeconomic and cultural level of the family, the lower the level of sexism of children and parents. In addition, Ayres et al. [45] explored the role of social support from family and friends as a protective factor in discrimination cases. Barry [46] also studied the influence of sexist parental attitudes. However, there were no studies that analyzed the possible relationship between family structure and sexism, considering the analysis of this relationship to be a differentiating element as provided by the study herein.

Lastly, it is worth mentioning that, according to the data analysis, students in the Colombian university show higher levels of ambivalent sexism than students in Spanish universities. Nevertheless, it should be noted that there are no statistically significant variables included in this study that could explain this phenomenon in the Colombian university, which is why other explanatory variables should be studied. Several causes have undoubtedly contributed to this situation. The adoption and enforcement of regulations aimed at combating GBV may have been a key element in this process. Thus, although these two countries have developed gender regulations, Spain has autonomously addressed the problems of women, preventing them from vanishing into the family category, as can be seen on several occasions in the Colombian gender regulations. Spanish has implemented tools such as the mandatory design and implementation of employment equality plans in companies with more than 50 workers (RDL 6/2019) or measures for the promotion of equality in the workplace, which amends Organic Law 3/2007 for effective equality between women and men; the specific regulation of co-responsibility in couples regarding childcare (RDL 6/2019) with measures for the promotion of equality in the workplace; the obligation to apply gender perspectives to all university programs (Organic Law 3/2007 of March 22); and the creation of specific courts to hear cases of violence against women (Organic Law 1/2004 on Comprehensive Protection Measures against Gender Violence), among others. Given all the aforementioned, we cannot affirm that the findings of this study can be generalized to other populations, given the influence of the education received and the experiences as well as other characteristics inherent to the person not contemplated in this study.

### Limitations and Future Research

This study have some limitations and it is worth pointing out, but reorienting those limitations back to the strengths of the study. Firstly, this is a cross-sectional study, so its results cannot be attributed to causality. Secondly, this is a multicenter study including three universities from two countries, these institutions are not nationwide representative. Therefore, it is possible that in other institutions, the students’ attitudes could be different of what was found here. Thirdly, non-probabilistic sampling methods were used for participant selection, which limits the representativeness of the sample and, consequently, the generalization of the results to the entire population and the sampling characteristic (university students in their final academic year) ignore other younger students who are more likely to have developed less consistent attitudes related to sexism. Finally, some participants refused to participate, so it could missed some important participants (i.e., victims).

It would be interesting in future research to conduct experimental studies that measure the change in sexism attitudes and assess this follow-up during the students’ years of study.

## 5. Conclusions

Despite the fact that the deep psychological roots of any type of violence remain an arcane, and more so in the case of violence against women, generalized beliefs and social *mores* seem to facilitate the acceptance, reduce the social visibility, and establish a benevolent tolerance of harmful behavior. The literature shows that gender education in the college environment contributes to making sexism and GBV visible among young people. So, gender education could have a positively influence in the value structure of students and helps them to trigger greater rejection of sexism and GBV. The relationship between sexist attitudes and gender violence suggests that training efforts to warn against sexism that are carried out in the university environment may lead to a lower prevalence of GBV. This research will aid these efforts by highlighting the inverse relationship between gender training and sexist beliefs.

The analysis of ambivalent sexism in university students in Colombia and Spain provides a realistic perception of young people’s attitudes. The results obtained in this empirical research help us to make comparisons with other studies as well as to analyze the level of consistency of our results, or the existing differences, and thus contribute to the existing field of research on the differential approach and the reduction of gender-based violence at both national and international levels. Making visible the roots of gender-based violence and the differential effects that arise in social relations is undoubtedly an input that leads to the construction of a democratic society in which women can fully exercise their rights.

Consequently, it is important to conduct studies that involve measuring the level of ambivalent sexism using the ASI instrument. The results of this type of research should favor the creation of didactic strategies that can be applied in different contexts, such as the educational sector. Thus, students will to be aware of their personal shortcomings so as to have a more holistic vision in their professional work. University courses should include content, values, and skills that promote attitudes against male violence. Students should receive training on gender, so that they integrate the gender perspective in their personal and professional life. In terms of practical implications, this research contributes to the study of the prevention and reduction of gender-based violence in Colombia and Spain to provide more appropriate responses to address social reality.

## Figures and Tables

**Table 1:** Characteristics of the total sample and by subgroups according to the home university.

	Total (*n* = 357) Mean (SD)/*n* (%)	UDES (*n* = 74) Mean (SD)/*n* (%)	URJC (*n* = 161) Mean (SD)/*n* (%)	US (*n* = 122) Mean (SD)/*n* (%)	*p*-Value ^c^
*Sociodemographic Variables*					
Age ^a^	22.27 (3.57)	23.46 (5.64)	22.21 (2.09)	21.62 (3.35)	**0.002**
Gender Identity ^b^					0.377
Female	235 (65.83)	43 (58.1)	110 (68.3)	82 (67.2)	
Male	118 (33.05)	31 (41.9)	49 (30.4)	38 (31.1)	
Prefer not to say	4 (1.12)	0 (0)	2 (1.2)	2 (1.6)	
Marital Status ^b^					0.581
Single	307 (86.24)	65 (87.8)	140 (87.5)	102 (83.6)	
Other	50 (13.76)	9 (12.2)	20 (12.5)	20 (16.4)	
Religious Identity ^b^					**0.000**
None and I don’t believe in God	109 (30.53)	5 (6.8)	70 (43.5)	34 (27.9)	
None, but I believe in God	63 (17.65)	22 (29.7)	27 (16.8)	14 (11.5)	
Catholic	157 (43.98)	41 (55.4)	50 (31.1)	66 (54.1)	
Other	28 (7.84)	6 (8.1)	14 (8.7)	8 (6.6)	
Household income level ^b^					0.188
Low	48 (13.48)	11 (14.9)	24 (14.9)	13 (10.7)	
Medium	281 (78.93)	61 (82.4)	126 (78.3)	94 (77.7)	
High	27 (7.58)	2 (2.7)	11 (6.8)	14 (11.6)	
Family situation ^b^					**0.000**
I have children	12 (3.36)	10 (13.5)	0 (0)	2 (1.6)	
My parents are separated or divorced	61 (17.09)	22 (29.7)	23 (14.3)	16 (13.1)	
My father or mother (or both) passed away	17 (4.76)	4 (5.4)	7 (4.3)	6 (4.9)	
I have problems regarding my family situation	16 (4.48)	3 (4.1)	4 (2.5)	9 (7.4)	
I don’t have problems regarding my family situation	251 (70.31)	35 (47.3)	127 (78.9)	89 (73.0)	
*Variables related to sexism*					
Hostile sexism ^a^	26.53 (12.73)	34.53 (13.16)	22.35 (11.45)	27.21 (14.63)	**0.000**
Benevolent sexism ^a^	23.69 (11.32)	32.45 (13.10)	21.12 (9.53)	21.79 (9.71)	**0.000**
Total ASI score ^a^	50.23 (22.90)	66.88 (24.16)	43.52 (19.02)	49.04 (21.96)	**0.000**
Gender training ^b^ [Yes]	157 (44.35)	48 (66.7)	55 (34.4)	54 (44.3)	**0.000**

Note: Mean and standard deviation were used for quantitative variables ^a^, and frequency and percentage for qualitative variables ^b^. ^c^ *p*-value refers to the comparison of means or frequencies among the different universities for each of the variables studied. *p*-values < 0.05 appear in bold.

**Table 2:** Mean comparison between the different Ambivalent Sexism Inventory (ASI) items based on the home university.

	UDES *n* = 74 Media (sd)	URJC *n* = 161 Media (sd)	US *n* = 122 Media (sd)	*p*-Value ^a^
*Hostile sexism (total score)*	34.53 (13.16)	22.35 (11.45)	27.21 (14.63)	0.000
Item 2	3.05 (1.73)	2.48 (1.73)	2.80 (1.87)	0.057
Item 4	3.61 (1.69)	2.84 (1.58)	3.32 (1.66)	**0.002**
Item 5	3.36 (1.85)	2.08 (1.47)	2.52 (1.60)	**0.000**
Item 7	3.28 (1.93)	2.01 (1.53)	2.57 (1.93)	**0.000**
Item 10	3.04 (1.74)	1.76 (1.31)	1.94 (1.44)	**0.000**
Item 11	3.12 (1.88)	1.74 (1.30)	2.16 (1.67)	**0.000**
Item 14	2.68 (1.63)	1.61 (1.05)	1.89 (1.38)	**0.000**
Item 15	2.78 (1.83)	1.61 (1.05)	1.93 (1.48)	**0.000**
Item 16	3.24 (1.71)	1.90 (1.34)	2.62 (1.69)	**0.000**
Item 18	2.96 (1.72)	2.04 (1.53)	2.79 (1.76)	**0.000**
Item 21	3.35 (1.78)	2.27 (1.58)	2.91 (1.91)	**0.000**
*Benevolent sexism (total score)*	23.69 (11.32)	32.45 (13.10)	21.12 (9.53)	**0.000**
Item 1	2.72 (1.73)	1.71 (1.75)	1.75 (1.37)	**0.000**
Item 3	2.66 (1.60)	2.04 (1.43)	2.02 (1.45)	**0.005**
Item 6	2.11 (1.63)	1.29 (0.92)	1.39 (1.19)	**0.000**
Item 8	3.38 (1.80)	1.67 (1.26)	1.81 (1.40)	**0.005**
Item 9	4.05 (1.81)	2.43 (1.83)	2.53 (1.91)	**0.000**
Item 12	3.04 (1.89)	1.52 (1.21)	1.53 (1.14)	**0.009**
Item 13	2.39 (1.72)	1.55 (1.22)	1.44 (1.09)	**0.000**
Item 17	2.97 (1.98)	2.07 (1.53)	2.23 (1.73)	**0.001**
Item 19	3.86 (1.72)	3.29 (1.67)	3.45 (1.65)	0.053
Item 20	2.08 (1.57)	1.41 (1.51)	1.51 (1.21)	**0.000**
Item 22	3.19 (1.88)	2.06 (1.46)	2.21 (1.53)	**0.000**
***Total ASI score***	66.88 (24.16)	43.52 (19.02)	49.04 (21.96)	**0.000**

^a^*p*-value refers to the comparison of means among the different universities for each of the variables studied. *p*-values < 0.05 appear in bold.

**Table 3:** Factors associated with sexism (total ASI score) for the total sample (*n* = 357).

Variable	OR_CRUDE_/(CI95%)	*p*	Model 1		Model 2	
			OR_ADJUSTED_/(CI95%)	*p*	OR_ADJUSTED_/(CI95%)	*p*
Age, years (centered on 21)	1.52 (0.85; 2.18)	**0.000**	0.74 (0.07; 1.40)	**0.030**	0.83 (0.27; 1.38)	**0.003**
Marital status [Single]	−7.12 (−14.00; −0.23)	**0.043**	−1.78 (−7.75; 4.19)	0.558		
Gender training [Yes]	4.93 (0.07; 9.77)	**0.047**	−0.52 (−4.64; 3.60)	0.805		
Gender Identity [Female]						
Male	18.23 (13.50; 22.96)	**0.000**	16.72 (12.53; 20.90)	**0.000**	16.92 (12.82; 21.02)	**0.000**
Prefer not to say	32.45 (11.51; 53.39)	**0.002**	28.16 (8.77; 47.54)	**0.005**	28.30 (9.59; 47.00)	**0.003**
Household income level [very low-low]						
Medium	−2.90 (−9.99; 4.20)	0.422	−0.15 (−6.13; 5.83)	0.960		
High-Very High	4.30 (−6.70; 15.29)	0.443	6.52 (−2.67; 15.72)	0.164		
Religious Identity [None and I don’t believe in God]						
None, but I believe in God	15.57 (8.959; 22.18)	**0.013**	9.80 (3.78; 15.83)	**0.002**	10.07; 4.21 (15.92).	**0.001**
Catholic	21.63 (16.41; 26.86)	**0.000**	16.17 (11.36; 20.99)	**0.000**	17.12 (12.40; 21.85)	**0.000**
Other	18.99 (10.21; 27.78)	**0.018**	13.63 (5.45; 21.81)	**0.001**	12.96 (4.94; 20.98)	**0.002**
Family situation [I have children]						
My parents are separated or divorced	−30.68 (−44.60; −16.77)	**0.000**	−6.54 (−20.51; 7.44)	0.358		
My father or mother (or both) passed away	−33.42 (−50.22; −16.62)	**0.000**	−10.64 (−26.50; 5.22)	0.188		
I don’t have problems regarding my family situation	−28.53 (−41.54; −15.52)	**0.000**	−3.60 (−17.36; 10.17)	0.608		
I have problems regarding my family situation	−29.35 (−46.16; −12.55)	**0.001**	−5.79 (−22.53; 10.96)	0.497		
Home university [UDES]						
University Rey Juan Carlos	−23.36;−29.29 (−17.43).	**0.000**	−16.96 (−22.97; −10.95)	**0.002**	−16.17 (−21.59; −10.75)	**0.000**
University of Sevilla	−17.83 (−24.05; −11.62)	**0.001**	−13.86 (−19.87; −7.85)	**0.020**	−13.04 (−18.62; −7.47)	**0.015**

Note: R^2^ (model 1) = 0.4259; *p* < 0.05. R^2^ (model 2) = 0.4135; *p* < 0.05. *p*-values < 0.05 appear in bold.

**Table 4:** Factors associated with hostile sexism and benevolent sexism for the total sample (*n* = 357).

Variable			Model 1 (Adjusted)	
	HS	BS	HS	BS
	OR_CRUDE_/(CI95%)		OR_ADJUSTED_/(CI95%)	
Age, years (centered on 21)	0.90 (0.50; 1.30) ***	0.62 (0.29; 0.96) ***	0.40;−0.02(0.82)	0.36 (0.02; 0.70) *
Marital status [Single]	−4.99 (−9.12; −0.87) *	−2.12;−5.53(1.28)	−1.24 (−5.02; 2.54)	−0.56 (−3.61; 2.48)
Gender training [Yes]	2.90 (0.00; 5.79) *	2.28;−0.10(4.67)	0.17 (−2.43; 2.78)	−0.60 (−2.70; 1.49)
Gender Identity [Female]				
Male	9.94 (7.08; 12.79)***	7.94 (5.56; 10.32) ***	9.22 (6.58; 11.86) ***	7.15 (5.03; 9.27) ***
Prefer not to say	23.76 (11.10; 36.43) ***	8.56;−2.05(19.16)	19.85 (7.58; 32.13) **	8.15;−1.74(18.04)
Household income level [very low-low]				
Medium	−2.23 (−6.46; 1.99)	−0.25 (−3.74; 3.25)	−0.74 (−4.50; 3.01)	0.64 (−2.41; 3.69)
High-Very High	−0.93 (−7.51; 5.65)	5.12 (−0.23; 10.47)	−0.65 (−6.46; 5.15)	6.70 (2.07; 11.33) **
Religious Identity [None and I don’t believe in God]				
None, but I believe in God	9.35 (5.37; 13.33) *	6.46 (3.15; 9.77) ***	6.68 (2.89; 10.48) ***	3.32 (0.26; 6.39) *
Catholic	12.09 (8.94; 5.24) ***	9.82 (7.21; 12.42) ***	9.32 (6.28; 12.35) ***	7.23 (4.80; 9.66) ***
Other	10.56 (5.24; 15.88) ***	8.57 (4.16; 12.97) ***	6.49 (1.32; 11.66) *	7.20 (3.03; 11.37) ***
Family situation [I have children]				
My parents are separated or divorced	−18.30 (−26.62; −9.98) ***	−12.09 (−19.02; −5.16) ***	−5.96;−14.81(2.88)	−0.06 (−7.17; 7.05)
My father or mother (or both) passed away	−21.06 (−30.98; −11.14) ***	−13.04 (−21.42; −4.66) ***	−9.29 (−19.25; 0.67)	−0.96 (−9.05; 7.12)
I don’t have problems regarding my family situation	−18.01 (−25.79; −10.23) ***	−10.54 (−17.02; −4.05) ***	−5.49 (−14.19; 3.21)	2.30 (−4.71; 9.31)
I have problems regarding my family situation	−15.25 (−25.30; −5.20) ***	−14.10; −22.48(−5.72) **	−3.67 (−14.26; 6.93)	−1.63 (−10.16; 6.90)
Home university [UDES]				
University Rey Juan Carlos	−12.19 (−15.79; −8.58) ***	−11.33 (−14.23; −8.44) ***	−7.28 (−11.07; −3.49) ***	−9.74 (−12.79; −6.69) ***
University of Sevilla	−7.33 (−11.11; −3.54) ***	−10.66 (−13.70; −7.62) ***	−4.13 (−7.92; −0.33) *	−9.99 (−13.05; −6.94) ***

Note: R^2^ (model 1-HS) = 0.3623; *p* < 0.05. R^2^ (model 1-BS) = 0.391; *p* < 0.05. *** *p* < 0.001 ** *p* < 0.01 * *p* < 0.05.

**Table 5:** Factors associated with sexism (total ASI score) depending on the home university.

Variable	UDES	URJC	US
	OR_ADJUSTED_/(CI95%)	OR_ADJUSTED_/(CI95%)	OR_ADJUSTED_/(CI95%)
Age, years ^a^	0.99 (−0.22; 2.20)	0.29 (−1.10; 1.69)	0.42 (−1.22; 2.07)
Marital status [Single]	−1.81 (−22.78; 19.16)	−4.09 (−12.01; 3.84)	0.39 (−9.31; 10.08)
Gender training [Yes]	−9.05 (−21.68; 3.57)	−1.50 (−7.27; 4.28)	3.20 (−3.92; 10.32)
Gender Identity [Female]			
Male	11.28 (−0.83; 23.39)	16.75 (11.19; 22.30) ***	21.50 (14.12; 28.88) ***
Prefer not to say	--	18.90;11.19 (22.30)	47.41 (7.24; 87.58) *
Household income level [very low-low]			
Medium	4.12 (−13.10; 21.34)	−2.45;−10.64(5.73)	−1.18 (−12.77; 10.40)
High-Very High	69.10 (4.44; 133.76) *	0.54 (−11.89; 12.97)	6.17 (−9.42; 21.76)
Religious Identity [None and I don’t believe in God]			
None, but I believe in God	22.09 (−1.52; 45.69)	14.80 (7.08; 22.51) ***	2.95 (−8.98; 14.88)
Catholic	18.97 (−3.49; 41.44)	18.97 (12.78; 25.16) ***	15.53 (7.44; 23.62) ***
Other	36.15 (6.79; 65.51) *	8.69;−0.75(18.13)	14.21;−3.47(31.89)
Family situation [I have children]			
My parents are separated or divorced	−10.76 (−35.04 (13.52)	--	−24.12 (−61.27; 13.03)
My father or mother (or both) passed away	−10.10 (−41.71; 21.51)	−9.09 (−25.53; 7.35)	−29.19 (−69.17; 10.79)
I don’t have problems regarding my family situation	−37.01 (−92.82; 18.80)	−7.69 (−25.64; 10.26)	−25.22 (−65.95; 15.51)
I have problems regarding my family situation	4.80 (−17.83; 27.43)	−3.27 (−10.92; 4.38)	−20.72 (−58.36; 16.92)

Note: R^2^ (UDES) = 0.3438; *p* < 0.05. R^2^ (URJC) = 0.3787; *p* < 0.05; R^2^ (US) = 0.4302; *p* < 0.05. ^a^ Age was centered on the median for each university. The median was 21 years for UDES, 22 years for URJC, and 20.5 years for US. *p* < 0.05. *** *p* < 0.001 ** *p* < 0.01 * *p* < 0.05.

**Table 6:** Factors associated with hostile sexism depending on the home university.

Variable	UDES	URJC	US
	OR_ADJUSTED_/(CI95%)	OR_ADJUSTED_/(CI95%)	OR_ADJUSTED_/(CI95%)
Age, years ^a^	0.51 (−0.16; 1.18)	−0.08 (−0.96; 0.79)	0.13 (−0.97; 1.23)
Marital status [Single]	1.54 (−10.07; 13.15)	−1.41 (−6.40; 3.58)	−1.55 (−8.04; 4.95)
Gender training [Yes]	−4.01 (−11.02; 2.99)	−0.46 (−4.09; 3.17)	2.64 (−2.13; 7.41)
Gender Identity [Female]			
Male	5.47 (−1.19; 12.14)	9.18 (5.70; 12.66) ***	12.55 (7.61; 17.50) ***
Prefer not to say	--	6.30;−7.99(20.59)	32.01 (5.10; 58.92) *
Household income level [very low-low]			
Medium	0.96 (−8.59; 10.51)	−2.80 (−7.90; 2.29)	−3.10 (−10.86; 4.66)
High-Very High	32.40 (−3.43; 68.22)	−3.06 (−10.85; 4.73)	−3.65 (−14.10; 6.79)
Religious Identity [None and I don’t believe in God]			
None, but I believe in God	13.10 (0.16; 26.05) *	9.81 (4.99; 14.62) ***	3.05 (−4.95; 11.04)
Catholic	9.22;−3.25(21.69)	10.81 (6.93; 14.69) ***	10.61 (5.19; 16.03) ***
Other	15.15 (−1.15; 31.44)	3.30;−2.62(9.22)	10.92;−0.93(22.76)
Family situation [I have children]			
My parents are separated or divorced	−9.92 (−23.36; 3.52)	--	−23.15 (−48.04; 1.74)
My father or mother (or both) passed away	−5.21 (−22.73; 12.30)	−7.91 (−17.50; 1.68)	−28.84 (−55.63; −2.06) *
I don’t have problems regarding my family situation	−26.45 (−57.34; 4.44)	−9.20 (−20.50; 2.09)	−19.91;−47.20(7.38)
I have problems regarding my family situation	−1.00 (−13.55; 11.55)	−3.57 (−8.37; 1.23)	−22.61 (−47.82; 2.61)

Note: R^2^ (UDES) = 0.2964; *p* = 0.077; R^2^ (URJC) = 0.3333; *p* < 0.05; R^2^ (US) = 0.4322; *p* < 0.05. ^a^ Age was centered on the median for each university. The median was 21 years for UDES, 22 years for URJC, and 20.5 years for US. *p* < 0.05. *** *p* < 0.001 ** *p* < 0.01 * *p* < 0.05.

**Table 7:** Factors associated with benevolent sexism depending on the home university.

Variable	UDES	URJC	US
	OR_ADJUSTED_/(CI95%)	OR_ADJUSTED_/(CI95%)	OR_ADJUSTED_/(CI95%)
Age, years ^a^	0.47 (−0.20; 1.15)	0.43 (−0.30; 1.15)	0.32 (−0.44; 1.09)
Marital status [Single]	−3.67 (−15.36; 8.02)	−2.61 (−6.74; 1.52)	1.87 (−2.63; 6.38)
Gender training [Yes]	−4.57 (−11.56; 2.41)	−1.24 (−4.24; 1.75)	0.99 (−2.29; 4.26)
Gender Identity [Female]			
Male	5.55 (−1.18; 12.27)	7.21 (4.33; 10.08) ***	8.57 (5.17; 11.97) ***
Prefer not to say	--	12.60 (0.77; 24.44) *	14.62;−4.02(33.26)
Household income level [very low-low]			
Medium	3.41 (−6.19; 13.00)	0.33 (−3.94; 4.60)	1.75 (−3.63; 7.13)
High-Very High	35.61 (−0.43; 71.65)	3.45 (−3.03; 9.94)	8.25 (1.22; 15.27) *
Religious Identity [None and I don’t believe in God]			
None, but I believe in God	8.41 (−4.74; 21.57)	4.97 (0.95; 8.98) *	0.51;−4.99(6.01)
Catholic	10.28 (−2.22; 22.78)	8.53 (5.33; 11.73) ***	5.60 (1.92; 9.27) **
Other	20.94 (4.56; 37.32) *	5.38 (0.46; 10.30) *	3.55 (−4.66; 11.76)
Family situation [I have children]			
My parents are separated or divorced	0.43 (−12.95; 13.80)	--	−0.77 (−18.02; 16.49)
My father or mother (or both) passed away	−4.42 (−22.04; 13.20)	−1.24 (−9.82; 7.34)	−0.01 (−18.58; 18.56)
I don’t have problems regarding my family situation	−9.14 (−40.20; 21.92)	1.46 (−7.90; 10.82)	−4.67 (−23.57; 14.24)
I have problems regarding my family situation	5.90 (−6.72; 18.52)	0.36 (−3.62; 4.35)	2.10 (−15.38; 19.58)

Note: R^2^ (UDES) = 0.3139; *p* < 0.051. R^2^ (URJC) = 0.3274; *p* < 0.05; R^2^ (US) = 0.3635; *p* < 0.05. ^a^ Age was centered on the median for each university. The median was 21 years for UDES, 22 years for URJC, and 20.5 years for US. *p* < 0.05. *** *p* < 0.001 ** *p* < 0.01 * *p* < 0.05.

## Appendix A. Ambivalent Sexism Inventory (Spanish Version)

1. Aun cuando un hombre logre muchas cosas en su vida, nunca podrá sentirse verdaderamente completo a menos que tenga el amor de una mujer (B)
2. Con el pretexto de pedir “igualdad”, muchas mujeres buscan privilegios especiales, tales como condiciones de trabajo que las favorezcan a ellas sobre los hombres (H)
3. En caso de una catástrofe, las mujeres deben ser rescatadas antes que los hombres (B).
4. La mayoría de las mujeres interpreta comentarios o conductas inocentes como sexistas, es decir, como expresiones de prejuicio o discriminación en contra de ellas (H).
5. Las mujeres se ofenden muy fácilmente (H).
6. Las personas no pueden ser verdaderamente felices en sus vidas a menos que tengan pareja del otro sexo (B).
7. En el fondo, las mujeres feministas pretenden que la mujer tenga más poder que el hombre (H)
8. Muchas mujeres se caracterizan por una pureza que pocos hombres poseen (B).
9. Las mujeres deben ser queridas y protegidas por los hombres (B).
10. La mayoría de las mujeres no aprecia completamente todo lo que los hombres hacen por ellas (H).
11. Las mujeres intentan ganar poder controlando a los hombres (H).
12. Todo hombre debe tener una mujer a quien amar (B).
13. El hombre está incompleto sin la mujer (B).
14. Las mujeres exageran los problemas que tienen en el trabajo (H).
15. Una vez que una mujer logra que un hombre se comprometa con ella, por lo general intenta controlarlo estrechamente (H).
16. Cuando las mujeres son vencidas por los hombres en una competencia justa, generalmente ellas se quejan de haber sido discriminadas (H).
17. Una buena mujer debería ser puesta en un pedestal por su hombre (B).
18. Existen muchas mujeres que, para burlarse de los hombres, primero se insinuuan sexualmente a ellos y luego rechazan los avances de éstos (H).
19. Las mujeres, en comparación con los hombres, tienden a tener una mayor sensibilidad moral (B).
20. Los hombres deberían estar dispuestos a sacrificar su propio bienestar con el fin de proveer seguridad económica a las mujeres (B).
21. Las mujeres feministas están haciendo demandas completamente irracionales a los hombres (H).
22. Las mujeres, en comparación con los hombres, tienden a tener un sentido más refinado de la cultura y el buen gusto (B).

Note: The letter B indicates that the article measures benevolent sexism and the letter H, hostile.

## Appendix B. Ambivalent Sexism Inventory (English Version)

1. No matter how accomplished he is, a man is not truly complete as a person unless he has the love of a woman.
2. Many women are actually seeking special favors, such as hiring policies that favor them over men, under the guise of asking for “equality.”
3. In a disaster, women ought to be rescued before men.
4. Most women interpret innocent remarks or acts as being sexist.
5. Women are too easily offended.
6. People are not truly happy in life without being romantically involved with a member of the other sex.
7. Feminists are seeking for women to have more power than men.
8. Many women have a quality of purity that few men possess.
9. Women should be cherished and protected by men.
10. Most women fail to appreciate fully all that men do for them.
11. Women seek to gain power by getting control over men.
12. Every man ought to have a woman whom he adores.
13. Men are incomplete without women.
14. Women exaggerate problems they have at work.
15. Once a woman gets a man to commit to her, she usually tries to put him on a tight leash.
16. When women lose to men in a fair competition, they typically complain about being discriminated against.
17. A good woman should be set on a pedestal by her man.
18. Many women get a kick out of teasing men by seeming sexually available and then refusing male advances.
19. Women, compared to men, tend to have a superior moral sensibility.
20. Men should be willing to sacrifice their own well being in order to provide financially for the women in their lives.
21. Feminists are making unreasonable demands of men.
22. Women, as compared to men, tend to have a more refined sense of culture and good taste.

Note: Glick et al., 2000 [36].
